# Leprosy postexposure prophylaxis with single-dose rifampicin: Nepalese dermatologist’s dilemma

**DOI:** 10.1371/journal.pntd.0009039

**Published:** 2021-04-08

**Authors:** Niraj Parajuli, Yogesh Poudyal

**Affiliations:** 1 Department of Dermatology & Venereology, National Academy of Medical Sciences, Bir Hospital, Kathmandu, Nepal; 2 Sarnath Skin Center, Shanti Path, Bhairahawa, Nepal; Universidade Federal do Para, BRAZIL

Nepal attained the status of leprosy elimination as a public health problem in 2010, however still struggling to sustain it. A recent World Health Organization (WHO) report has shown an increase in new case detection from Nepal in 2019 (3,844 new cases) which is an alarming situation [1]. In an effort to sustain the elimination status and reduce transmission, leprosy postexposure prophylaxis (LPEP) with single-dose rifampicin (SDR) has been implemented in some districts as a part of the national leprosy control program.

The differences of opinions among leprosy experts on LPEP has created certain dilemmas. Although SDR is the only chemoprophylaxis recommended by WHO, we would like to share some of our dilemmas with LPEP with SDR with respect to these certain perspectives:

1. Contact definition;
2. Adherence to WHO guidelines in initiating SDR;
3. Steps used to rule out tuberculosis and possibility of rifampicin resistance;
4. Dose of rifampicin.

A single-centered, double-blind, cluster-randomized, placebo-controlled trial (COLEP) study showed that SDR had 57% protection to the contacts of the index case [2]. It showed that SDR LPEP provided a significant protection for contacts of paucibacillary (PB) and single-lesion leprosy but did not provide significant protection for contacts of multibacillary (MB) leprosy index cases. It also showed significant protection from developing leprosy in the social contacts but not in the immediate family members during the first 2 years. However, a follow-up of the same study at 4 and 6 years showed no significant protection after the first 2 years [3]. Regardless of the disparities on the treatment outcome among subgroups, the overall outcome of COLEP study was still used as an evidence by WHO to recommend SDR as LPEP for the contacts [4]. Leprosy experts have expressed different opinions regarding SDR and are still failing to find a common ground [5,6]. While LPEP intervention provides an opportunity for an increased contact examination, it is the use of oral rifampicin that is prompting much of the discussion.

## Contact definition

Definition for contacts varies among different countries depending on the criteria set forth [7]. Nepal’s LPEP program has put forth a criterion for contact definition. Nepal is a geographically diverse country with differences in the population densities and the landscape from the mountains in the north which is less populous to the more populous flat lands of Terai in the south. Similarly, the population density in crowded metropolitan is in contrast to sparsely populated rural regions. Our concern is whether a single definition for social and neighbor contact takes into consideration the geographical and population density differences of Nepal. If SDR is found to be effective only among social contacts and not among the immediate household contact, should we still provide SDR to the household contacts? Even in the COLEP study, the leprosy proportion among contacts remains the same at the time of enrolment and at 4 years of SDR follow-up which needs to be explored.

## Adherence with WHO guidelines on initiating SDR

According to the WHO guidelines, SDR should be implemented after fulfilling the 2 conditions, one of them being the consent of the index case to disclose their disease status. However, multiple studies from Nepal have suggested a significant level of stigma associated with leprosy in most parts of the country [8–10]. Despite continuous efforts to reduce the social stigma associated with leprosy through community awareness, less progress has been made so far. So, is it possible to implement LPEP with SDR properly in such circumstances? Our concern is that if we will not be able to provide adequate counseling during SDR, it could instead provide false sense of long-term protection from leprosy and patients may ignore the early symptoms of leprosy and not visit health centers for checkups. Therefore, the healthcare staffs must be trained properly before starting such intervention; otherwise, it might be disastrous.

## Steps used to rule out tuberculosis and rifampicin resistance

Another confusion is with respect to criteria for ruling out tuberculosis (TB) and rifampicin resistance. LPEP describes certain features to rule out TB. An expert consensus concluded only a minimal risk of inducing rifampicin resistance in TB with SDR but some experts are still skeptical [5,11]. It has been found that 15% to 38% of all TB cases may not show any symptoms, including extrapulmonary and childhood TB [11]. Extrapulmonary TB represents one-third of the total cases, and childhood TB constitutes 5.5% of all total TB cases in Nepal and not to mention the unrecorded cases [12]. A 2.2% of multidrug resistance to antitubercular treatment (ATT) has been reported in the newly diagnosed TB patients in Nepal [12]. Even in leprosy, a WHO collaborative multicentric study from 11 countries showed that 2% of newly diagnosed cases were resistant to rifampicin along with other antimicrobials [13]. So, what prevalence of rifampicin resistance would be a threshold when we should be cautious regarding its administration in the mass population for LPEP?

## Dose of rifampicin

Newer trials like Post Exposure Prophylaxis for Leprosy (PEOPLE) in the Comoros and Madagascar uses double dose of rifampicin (20 mg/kg) to the contacts [14], and PEP ++ combines rifampicin with either clarithromycin or moxifloxacin (a fluoroquinolone with potential side effects) for 3 once monthly doses [15]. Both of these studies use different doses and/or combinations of rifampicin. So, would it be worthwhile to wait for the results of such studies before implementing SDR as LPEP?

Leprosy behaves differently in its spectrum with short incubation period in PB leprosy to up to years in MB leprosy. With new insights coming from the MALTALEP trial [16] (which included only the household contacts and first neighbors and showed the level of protection of only 42% as compared to 57% in COLEP study) and the follow-ups of the COLEP study [3], it would certainly be beneficial to get an updated recommendation of WHO on LPEP with SDR, which has a huge impact on formulating the national policies.

A recent published study showed that multidose of postexposure prophylaxis for chemoprophylaxis may be necessary to control the infection with subclinical leprosy to further prevent disease and decrease the transmission [17]. This article entirely brings a new discussion of whether we should even implement SDR as LPEP, further supporting our argument on the dilemmas for its implementation in the national leprosy control programs.

Dr. Lockwood and colleagues had even requested the WHO Global Leprosy program for further research and wait before implementing SDR in the national leprosy control programs. There is no doubt that we need better intervention to further reduce the transmission of leprosy and progress toward zero leprosy. But, will such regimen with limited studies on its proven benefits and many disagreements within the fraternity be the right strategic intervention to achieve the targets set by the national leprosy programs? Should we rather consider on intensifying the contact tracing and examination programs while awaiting for an expert consensus on LPEP? Further expert discussions might be necessary to expand our knowledge and produce clarity on our dilemmas with regard to LPEP with SDR.
